# Ultrafast, Non-Productive, Deprotonation of the Excited Flavylium Cation in Delphinidin-3-O-Rutinoside

**DOI:** 10.3390/molecules31203645

**Published:** 2026-10-13

**Authors:** Florian Bartonitz, J. Luis Pérez Lustres, Karsten Heyne

**Affiliations:** Institut für Experimentalphysik, Freie Universität Berlin, Arnimallee 14, D-14195 Berlin, Germany; fbartoni@zedat.fu-berlin.de (F.B.);

**Keywords:** femtosecond, spectroscopy, transient absorption, flavonoids, anthocyanins, anthocyanidins, proton transfer to solvent

## Abstract

Anthocyanins are widespread natural pigments responsible for the vibrant colors of fruits and flowers and play key roles in photoprotection. Despite their biological significance, the interplay between excited-state proton transfer (ESPT) and ultrafast relaxation remains poorly understood. Here, we investigate the ultrafast excited-state dynamics of delphinidin-3-O-rutinoside (D3R) in aqueous solution at pH 2—where the protonated flavylium cation dominates—using femtosecond broadband transient absorption spectroscopy. Excitation at 525 nm induces rapid spectral changes on the sub-picosecond timescale connected with sub 50 fs deprotonation in the excited state. Global analysis reveals two dominant processes: a 0.12 ps component assigned to ultrafast proton transfer from the excited flavylium cation to the solvent, forming the electronically excited quinoidal base (QB). This is followed by a 0.47 ps component characterized by strong bleach recovery, representing the primary relaxation pathway back to the ground state of at least 70%. Partial germinal reprotonation occurs upon electronic relaxation to the ground state. A mixture of vibrationally excited D3R and QB results. A characteristic time scale is around 3 ps. Reprotonation of QB occurs on the ps–ns timescale via diffusion-controlled processes. The overall process enables rapid dissipation of excitation energy, providing a mechanistic basis for the photoprotective function of anthocyanins in biological systems.

## 1. Introduction

Anthocyanins (AC) are a large class of water-soluble natural pigments [1]. They derive from parent flavonoid anthocyanidin chromophores, which are etherified with one or more sugars. AC play an important role in human diet, as they are found in high concentrations in plants and fruits, to whom they confer vivid blue, purple, red and orange colors [1,2,3,4,5]. They fulfill a number of functions, among which protection against UV radiation [6], cancer [7] and oxidative stress [8,9,10,11], can be underlined as most important.

The first reports on ACs were published more than a century ago and were reviewed by Willstätter and coworker in Reference [12]. At that time, ACs were already isolated from plants, synthesized [12,13] and elucidation of their basic molecular structure was completed. A large number of those compounds is now known, see Scheme 1, as well as their distribution among plants and fruits. Interestingly, ACs may exist in various protonation states but understanding of acid-base equilibria is complicated by the various protonation sites and competing hydration and tautomerization equilibria which lead to several possible neutral and anionic acid-base forms, Scheme 2.

Deprotonation of the cationic form leads either to the neutral quinoidal or (upon hydration) to the hemiketal bases (carbinol pseudobase), see Scheme 3. This was studied for malvidin, in which an equilibrium ratio of quinoidal-to-hemiketal base of 1:100 has been established [14]. The hemiketal base is colorless and further produces, upon opening of the central C-ring, the chalcone forms. The latter show yellow to green color and may exist in *cis* and *trans* isomers, Scheme 3. Thus, as the pH increases, populations of the neutral bases start to evolve in time hindering strongly ${\mathrm{pK}}_{a}$ determination by spectrophotometrical methods [15]. The process is reversible: acidification of the neutral solution brings the equilibrium back to the protonated form, i.e., no degradation occurs. Furthermore, conversion between quinoidal and carbinol bases does not occur directly but through the protonated form [14,16].

This scenario is further complicated by copigmentation, dimerization [17] and metal complexation equilibria, which alter the behavior of ACs depending on concentration and composition of the medium. For instance, it has been proposed that copigmentation hinders formation of the colorless carbinol base by hydration. Thus, and in contrast to aqueous solution, the colorful quinoidal base of malvidin-3-O-glucoside results in it being the major neutral pigment in red wine at pH values around 3.6 [15].

Photoacidic behavior of ACs was proposed by Maçanita et al. in References [18,19]. The work, based on malvidin and model compounds, concludes ultrafast adiabatic proton transfer as deactivation mechanism in water. In these experiments time-resolution was limited to picoseconds in fluorescence measurements, and to nanoseconds in flash-photolysis experiments. Important findings were an excited-state proton-transfer in the excited state of AC (AC*), and a product formation detected on the nanoseconds time-scale at pH 3. This product was assigned to the quinoidal base (QB) in the electronic ground state with an absorption band around 600 nm. At later stages, QB reprotonates by a diffusion-controlled reaction. The general kinetic behavior of ACs or derivatives were described [20], but the exact timing of the proton-transfer, the involved electronic states and the extent of the reaction were still not specified and quantified. Here we investigate the excited-state proton-transfer process with improved time resolution by fs broadband transient absorption in the UV–Vis spectral range, so that both excited-state deprotonation and ground-state reprotonation can be traced continuously with a single technique. This provides further details about photoinduced formation of the quinoidal base. Ultrafast excited-state decay competes efficiently with proton-transfer to solvent and enables rapid dissipation of light energy, consistent with ACs protecting against radiation [18,19]. Thus, proton-transfer to solvent turns out to be a side product of the ultrafast internal conversion, as only a minor fraction of the initially excited population remains deprotonated on the early nanosecond timescale.

## 2. Results

Linear absorption spectra of D3R were measured in aqueous solution at pH values ranging from about 1 to 10. The spectra are stable at pH values below 3. At higher pH values UV–Vis spectra are known to evolve in time. This effect originates from a series of slow, reversible hydration equilibria affecting neutral and anionic forms. These processes may lead to opening of the central pyrane ring. It is important to note that the process is reversible, that is, nearly colorless neutral solutions become purple upon acidification. Also structural stability of the AC is better than that of the corresponding anthocyanidin as described by many authors before [12]. Therefore, UV–Vis spectra of the D3R anthocyanin were measured shortly after sample preparation to minimize spectral distortion due to the evolving equilibria. This series of data was analysed globally to obtain the characteristic ${\mathrm{pK}}_{a}$ values of D3R and the optical spectra assigned to each protonation state. Spectra of the cationic, neutral, anionic forms and another form we call dianionic form are shown in Figure 1B. The fitted ${\mathrm{pK}}_{a}$ values are 3.89±0.02, 6.89±0.02 and 8.90±0.02, corresponding to the D3R cation-neutral, neutral-anion and anion-dianion acid-base equilibria, respectively [21].

The cationic form shows strong absorption at about 510 nm in an acidic aqueous solution. Deprotonation leads to the neutral form, which is almost colorless. The so-determined spectrum of the neutral form is understood as a characteristic early spectrum of all neutral forms, namely quinoidal, hemiketal (also known as carbinol) bases and cis/trans chalcones, with time-dependent concentrations. As the absorption coefficient is small compared to the protonated and anionic forms, high contributions of carbinol and chalcone forms are concluded. Further deprotonation, at pH values beyond 7, forms the anionic form absorbing weakly at 575 nm. The absorption spectra of the dianionic form is almost indistinguishable from the anionic one in the Vis range, but exhibits stronger absorption in the UV at 350 nm.

Transient absorption spectroscopy of D3R at pH = 2 shows fast spectral evolution, Figure 2. At 50 fs delay, negative contributions of bleaching signal around 510 nm, stimulated emission (SE) around 600 nm and 700 nm, as well as positive excited-state absorption (ESA) signals around 380 nm are observed. The ESA signals decay within 0.5 ps to about half its strength, with less signal changes around 700 nm, and a rise of a small positive signal around 600 nm. Earliest dynamics up to 0.2 ps are characterized by a pattern change of SE between 570 and 770 nm, while bleaching and ESA remain essentially the same. On this time-scale the strong SE contribution around 600 nm decays, while the small SE contribution around 700 nm stays nearly constant. During the next 2 ps all bands decay, giving rise to a broad product absorption extending to the red of 550 nm. Bleach decays at this stage to about 30% of its initial value, indicating efficient back-reaction to the parent D3R state. No additional information from anisotropy measurements was gained.

Comparison of the bleaching signal at delay times longer than 1 ns with delay times at 3 ps shows a loss of product absorption of about ≈90% on this time-scale.

Transient spectra recorded in the 2 ns timescale are shown in Figure 3. The pump-probe polarization was set to magic angle, so that time-evolution is not distorted by rotational diffusion. The spectra at 3 ps delay contain a product band with maximum at 590 nm, bleaching band at 510 nm and some residual ESA at 380 nm, similar to the spectrum at 2 ps delay in Figure 2. We observed decay time constants of 3.2 ps, 439 ps, and 1813 ps (see Supporting Information).

## 3. Discussion

Transient absorption data were analyzed with the help of global analysis [22,23]. For this purpose, full datasets measured in various time-scales were analyzed globally with a basis-set of functions defined by four exponential decays with decay times $\tau_{1-4}$, a non-decaying component and further terms accounting for the coherent contribution to transient absorption at early time. The global fit is of very good quality (see Supporting Information), and yields decay times of 0.12 ± 0.02, 0.47 ± 0.04, 1.84 ± 0.08 and 2.69 ± 0.09 ps.

Transient data in Figure 2 and Figure 3 show clear ESA below 400 nm indicating population in the excited state. Negative signals around 510 nm reflect bleaching band contributions of D3R, while red-shifted negative signals are attributed to SE. Positive signals from 550 nm to 750 nm are assigned to QB ground-state product absorption or D3R hot ground-state absorption.

The spectral distributions of the various ultrafast decaying components are shown as Decay-Associated-Difference Spectra (DADS) in Figure 4. Here, the longest time constant, indicated as Offset, on the picosecond timescale (purple line in Figure 4) shows two weak contributions. The positive signal around 600 nm indicates the photoproduct QB [18], while the negative signal around 510 nm reflects the absorption loss of D3R molecules undergoing a photoreaction to QB. By comparing the bleaching signal strengths at 50 fs and around 2 ps we can estimate the QB quantum yield to about 2% to 5%. The fastest observed dynamics occur with 0.12 ps decay time. In this DADS, we see no bleaching signal supporting dynamics only in the electronic excited state. The strong negative signal in the range from 580 nm to 680 nm and a weak negative signal in the range from 700 nm to 750 nm should reflect dual SE contributions, i.e., SE 1 and SE 2, respectively. The positive contribution at about 500 can only be interpreted as a decay of ESA overlapping the bleach band. Otherwise, an increase of bleach slower than the instrument response function shall be concluded, which seems unphysical. This positive signal around 500 nm indicates a loss of ESA, while around 380 nm a negative signal should indicate a rise of ESA. This could be interpreted as a blue-shift of the ESA or a decay of one ESA (ESA 1) and the rise of another ESA (ESA 2). In Figure 2 we observe an isosbestic point at 415 nm at early delay times, supporting a transition from ESA 1 to ESA 2. The linear absorption spectra shown in Figure 1B reveal a dramatic decrease in oscillator strength by a factor of about 10 during the transition from the protonated D3R form to the neutral form QB. Thus, it seems plausible to assign the stronger ESA 1 and SE 1 to D3R* and ESA 2 and SE 2 to QB*. However, other explanations are also possible. We assume D3R to form hydrogen bonds with water molecules in a water solvent shell giving rise to different fast fluctuating structural arrangements. Upon photoexcitation the excited state dynamics are expected to differ for conformations where the proton is transferred to a strongly hydrogen-bonded water molecule or to a weakly bounded one. Proton transfer to water solvent was reported to show decay times of about 50 fs and in the hundred fs time-scale in line with our results [24]. We assign the decay time of 0.12 ps to the slower proton transfer to the water solvent.

The dual SE signals is known from excited-state proton-transfer reactions with a protonated form and a red-shifted deprotonated form [25]. In analogy, we assign the SE around 600 nm to the protonated form (D3R*) and the SE around 700 nm to the deprotonated form (QB*). Deprotonation of D3R and QB formation results in a change of the π-electronic system shifting the electronic transitions into the UV range, reducing dramatically the overall oscillator strength in the visible range (see Figure 1B). Upon photoexcitation we observed a significant reduction in oscillator strength for the positive signals in the visible spectral range (Figure 2) supporting deprotonation in the ES. This points to nearly instantaneous ES deprotonation for some of the D3R* molecules, probably those with strong hydrogen bonds to water molecules, while for weaker hydrogen bonded D3R* molecules a time constant of 0.12 ps is simulated. Here, we modeled the dynamics with a sum of exponentials, but other models such as a distribution of decay times seem also meaningful. We assign our fastest decay time of 0.12 ps to excited-state proton-transfer from D3R* to QB* and to relaxation of QB*. Note, on this time-scale the proton is transferred to adjacent water molecules and is able to transfer back if the ES decays.

The next decay time is observed with $\tau_{2}=0.47\;ps$. The DADS (red line in Figure 4) show significant decay (>60%) of the ESA at 380 nm and bleaching recovery around 510 nm. This significant decay of the bleaching signal and ESA 2 demonstrates the decay of the ES to the ground state. On this ultrafast time-scale we expect some protons become solvated and spatially separated from D3R leading to formation of deprotonated QB, while other protons solvate and reprotonate to D3R. The excess energy should be redistributed over vibrational modes of D3R and QB resulting in a vibrationally excited D3R’ and QB’ with red-shifted ground-state absorption. The negative shoulder around 560 nm, and the small negative signal around 700 nm might indicate the rise of these ground-state products. We found two additional time constants in the ultrafast timescale with decay times of $\tau_{3}=1.84\;ps$ and $\tau_{4}=2.69\;ps$. While the DADS of $\tau_{3}$ exhibits signs of ESA at 380 nm and of SE around 700 nm, in the DADS of $\tau_{4}$ both signal contributions are absent. We assign $\tau_{3}$ to a slower decay of QB*, and D3R* to ground-state products, explaining the absence of positive signals above 400 nm. In contrast, in the DADS of $\tau_{4}$ positive signal contributions are visible in the range from 400 nm to 680 nm, with a superimposed negative signal around 500 nm. We interpret the positive signal as ground-state photoproduct and the negative signal as bleaching. We expect two different ground-state products, a vibrationally excited D3R’ and a vibrationally excited QB’ which are both formed with 0.47 ps and 1.84 ps. Note, a proper separation between reaction pathways of D3R* and QB* is hampered by the drastically different oscillator strengths of both forms.

The QB transient spectra can be used to estimate the excited-state $pK_{a}^{*}$ value of D3R. The 00 transitions from $S_{0}$ to $S_{1}$ of the D3R and QB forms are approached as 551 nm and 659 nm, respectively. These are average transitions energies from the ground-state absorption and SE maxima. The latter could only be guessed from the rapidly evolving transient spectra. By applying the Förster cycle $\Delta pK_{a}=\frac{\Delta E}{2.303RT}$ and using a ground-state $pK_{a}$ value of 3.8, an acidity constant $pK_{a}^{*}$ of −2.3 results for the excited state. Here, ΔE is the energy difference between the electronic transition energies of the acid and its conjugate base, *R* is the universal gas constant, and *T* is the absolute temperature [26,27]. This low value is compatible with an ultrafast deprotonation reaction under solvent control (Figure 5, see also Supporting Information).

## 4. Materials and Methods

### 4.1. Sample Preparation

A total of 1.2 mg of delphinidin-3-O-rutinosidechloride (D3R) (powder) from ROTICHROM (Carl Roth GmbH & Co., KG, Karlsruhe, Germany) was dissolved in a 2.5 mL solution of perchloric acid (70%, ROTH and milliQ water). A pH value of 2 was prepared for all measurements. pH was determined with a pH-Meter (FiveEasy, Mettler Toledo, Switzerland) equipped with a combined electrode (InLab Micro, Mettler Toledo, Switzerland). pH calibration was done with technical buffer solutions for pH 7.00, 4.01 and 9.21 (InLab, Mettler Toledo). The final solution was in-line filtered (0.45 μm pore size, NeoLab) prior measurement. D3R samples showed maximal absorption of 0.3 at 510 nm in a 0.2 mm cell (Starna, UK). Concentration was so adjusted to prevent aggregation, which results in slowly increasing pump scattering in the transient absorption signal. Samples for linear absorption spectroscopy were prepared in the same way but diluted by a factor of 2 and measured in 1 mm cells (Starna).

### 4.2. Spectroscopic Methods

Linear absorption spectra were scanned on a Shimadzu UV-3101PC spectrophotometer (Shimadzu Corporation, Kyoto, Japan) and in 1 mm Spectrosil (Starna) cells. Transient absorption measurements in the Vis range were performed on a home-built setup as described in Reference [28]. Basically, fs pulses were derived from a Clark MXR CPA 2001 Ti:Sa laser system (Clark-MXR, Inc. in Dexter, Michigan, USA). The fundamental beam was divided to pump two home-built non-collinear optical parametric amplifiers [29], which were tuned to the same wavelength of 525 nm and used for optical pumping and continuum generation on 1 mm fused silica or monocrystalline CaF_2_ plates (Korth Kristalle GmbH, Altenholz-Kiel, Germany). The instrument response function is about 70 fs and the spectral coverage ranges from 350 nm to 750 nm. A flowing cell (0.2 mm internal thickness and 0.2 mm thickness for the front window, fused silica Spectrosil, Starna) was used to ensure fresh sample conditions after every pump pulse. Transient absorption measurements were performed with independent signal and reference spectrographs. Referencing was done for single pulses, hundred shots were averaged for every time delay and typically eight scans were further averaged to improve signal-to-noise. Pump energy was 0.5 μJ and the beam was focused to a spot size of about 150 μm. Pump-probe parallel, perpendicular and magic angle polarizations were measured. Stationary UV–Vis spectra were measured before and after the transient absorption measurements, and no indication of photodegradation was observed.

## 5. Conclusions

We have performed ultrafast Vis-pump Vis-probe measurements to trace ultrafast excited-state proton-transfer in D3R in water. Global analysis of our data reveals four decay times of 0.12 ± 0.02, 0.47 ± 0.04, 1.84 ± 0.08 and 2.69 ± 0.09 ps in the ultrafast time-range and decay times of about 400 ps and 1.8 ns the longer time-range. Upon photoexcitation we observed a significant reduction in oscillator strength for the positive signals. The dominant signal is the bleaching signal of D3R with a clearly smaller shoulder at longer wavelengths and a positive signal of excited-state absorption around 380 nm. This drop in oscillator strength is also observed upon deprotonation of D3R and formation of the neutral form QB (see Figure 1B). The decay associated difference spectra (DADS) provide spectral information on the dynamics. The fastest decay time of 0.12 ps shows no bleaching contribution and is thus interpreted as purely dynamics on the electronic excited state (ES). Here, we observed four spectral contributions, two of them assigned to excited-state absorption (ESA), ESA 1 at 380 nm and ESA 2 at 500 nm, while the other two contributions are assigned to stimulated emission (SE), SE 1 around 600 nm and SE 2 around 720 nm. We tentatively assigned ESA 1 and SE 1 to the protonated D3R* in the ES, and ESA 2 and SE 2 to the deprotonated QB* in the ES. The loss of D3R* population and rise of ESA 2 is interpreted as a deprotonation of D3R* to QB* with 0.12 ps. Since the overall signal change around 380 nm is below 30% within the first 0.3 ps, we assume that a major part of the excited molecules deprotonate within 50 fs, while a smaller fraction deprotonates with 0.12 ps. Solvation dynamics of water takes place with sub 50 fs, 120 fs, and 880 fs time constants, matching the excited-state decay times observed in D3R [30]. Thus, it seems plausible that water solvation dynamics governs the proton transfer of D3R in the ES, e.g., via fluctuations of hydrogen bonds and donor-acceptor distances.

The major contributions (≈70%) of the excited-state signals decay with a time-constant of 470 fs to the ground state. We observe no effect upon solvent deuteration, since the dynamics are nearly identical for measurements in H_2_O and D_2_O (see Supporting Information). With the decay of the ES we observe the rise of a product band that first undergoes some spectral changes and later on matches the QB absorption on the time-scale of several picoseconds to nanoseconds. We interpret the rise of the product band as either vibrationally excited D3R’ or vibrationally excited QB’.

Since the deactivation of the ES is very fast and efficient and probably coupled with a reprotonation process we propose a conical intersection between ES and ground state enabling this fast process, as observed in other systems [25]. Since, no hard evidence exists for reprotonation with a time constant of 0.47 ps further experiments have to be performed to support these interpretations.

A smaller fraction of D3R* and QB* decays to D3R’ and to the QB’ ground-state product with a time constant of 1.84 ps, followed by vibrational cooling of D3R’ and QB’ to D3R and QB with a time constant of 2.69 ps. In the ground-state reprotonation of QB occurs on the ps and ns timescale.

The so-obtained QB product spectrum matches well both the Oenin transient spectra recorded by flash-photolysis and the pH-jump (1→5.9) difference spectra [18]. Therefore, the QB spectrum is assigned to the quinoidal base QB only. A QB product quantum yield of 2–5% is estimated. QB is formed within few picoseconds and decays further by diffusion-controlled protonation from the bulk solvent. The latter takes place on the ns timescale with a loss of QB product by about 90%.

In summary, ultrafast excited-state proton-transfer to solvent is observed for D3R in the electronic excited state. QB* is formed within 50 fs and a 120 fs time constant. The major fraction goes back to the electronic ground state on a 500 fs timescale. Only small product signals are observed, probably because of the strongly reduced oscillator strength of QB. The bleaching signal gives an upper limit of 30% for the reaction quantum yield of QB. The excitation energy is probably distributed over vibrational modes in the ground state leading to a vibrationally excited QB’ or D3R’. Our measurements do not allow a separation of these two ground-state species, because of their distinct oscillator strengths. The ultrafast transition from excited to ground state occurs probably via a conical intersection. Reprotonation takes place upon D3R’ formation during the transition from the excited to the ground state, or for QB’ with decay times of 2.69 ps, and about 500 ps and 1 ns. The photoproduct signal decays by about 90% on the nanosecond time scale, giving a maximal quantum yield of about 3% for QB. D3R converts more than 95% of excitation light energy around 510 nm into heat on a nanosecond time-scale. This wavelength range provides peak intensities of solar light on earth and can provide radiation damage on fruits and plants. With D3R the absorbed light is efficiently dissipated into heat, probably protecting surrounding (bio)molecules from radiation damage. We expect this mechanism to be one of the biological functions of anthocyanins in fruits.

## Figures and Tables

**Scheme 1:**
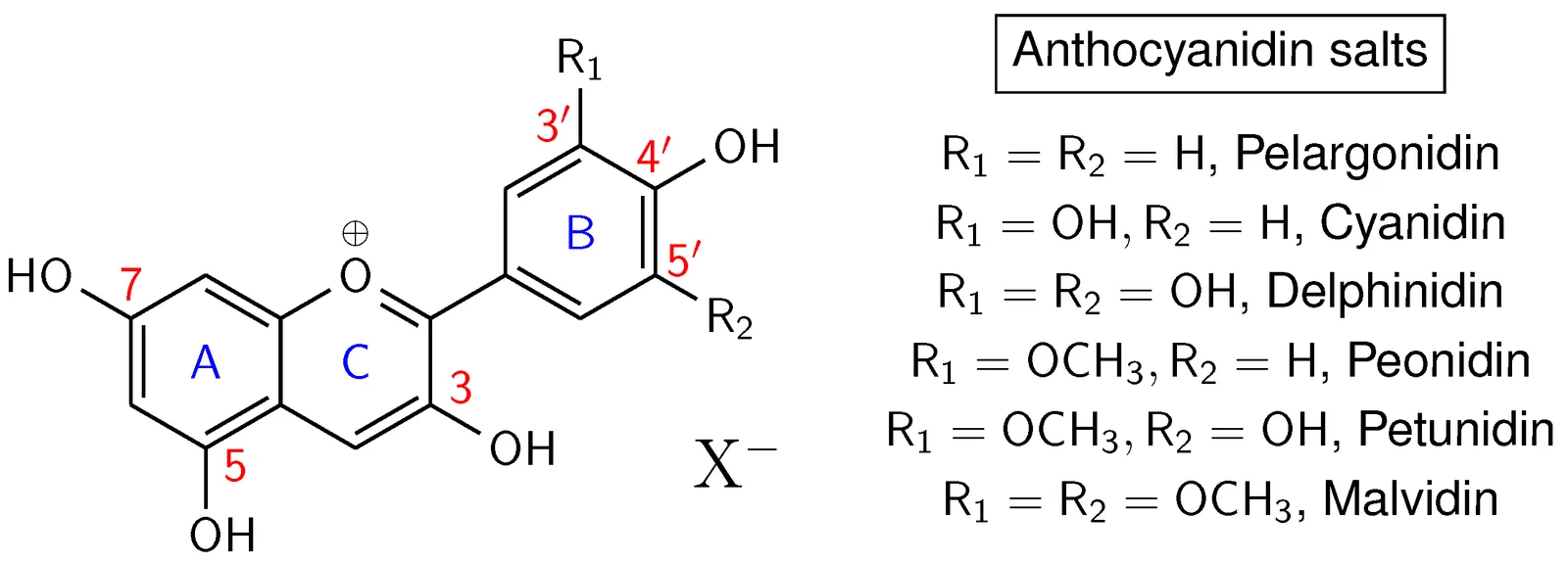
Chemical structure of the most abundant anthocyanidins. Positions of functional groups are highlighted in red. Blue capital letters mark typical naming of the three aromatic rings. The cationic form, with a formal positive charge on the pyrane oxygen (ring C) at position 1 is shown.

**Scheme 2:**
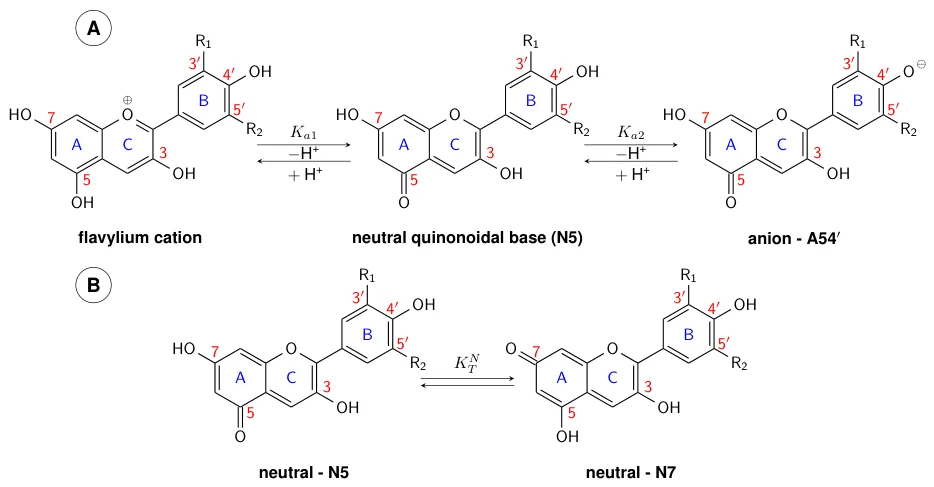
First two acid-base equilibria of ACs (panel (**A**)) and possible deprotonation sites of the flavylium cation linked by a tautomerization equilibrium in water (panel (**B**)).

**Scheme 3:**
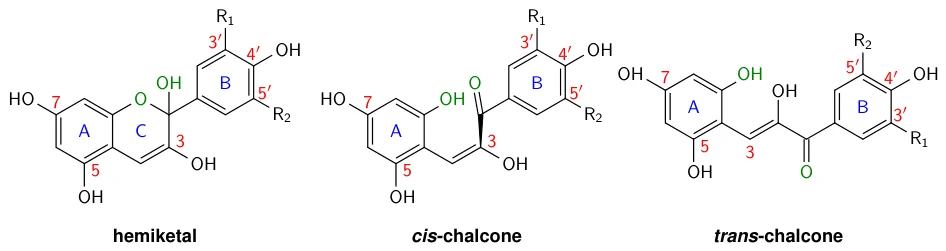
Hydration of the neutral form at position 2 gives rise to the hemiketal or carbinol base form (**leftmost structure**). Further opening of the central ring C forms the *cis* (**middle**) and *trans*-chalcones (**right**). All three forms are essentially colorless. The oxygen atom and the hydroxyl group involved in the ring opening process are highlighted in green for all three structures.

**Figure 1:**
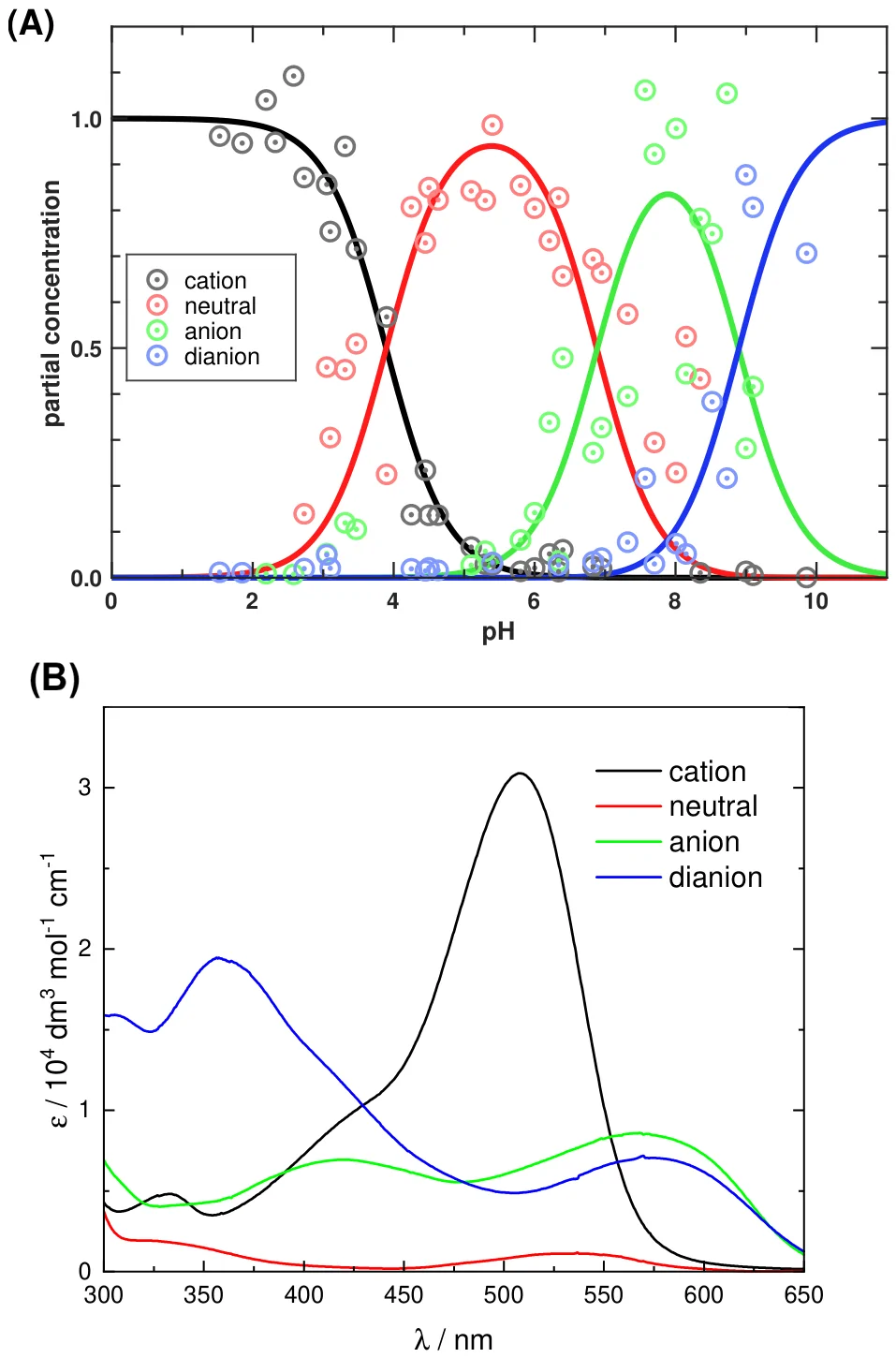
UV–Vis spectral contributions of the various acid-base forms of D3R at different pH values (**A**), as obtained by global analysis of pH-dependent spectra. This indicates four acid-base species for D3R in aqueous solution. The characteristic UV–Vis absorption spectra of these four species are shown (**B**).

**Figure 2:**
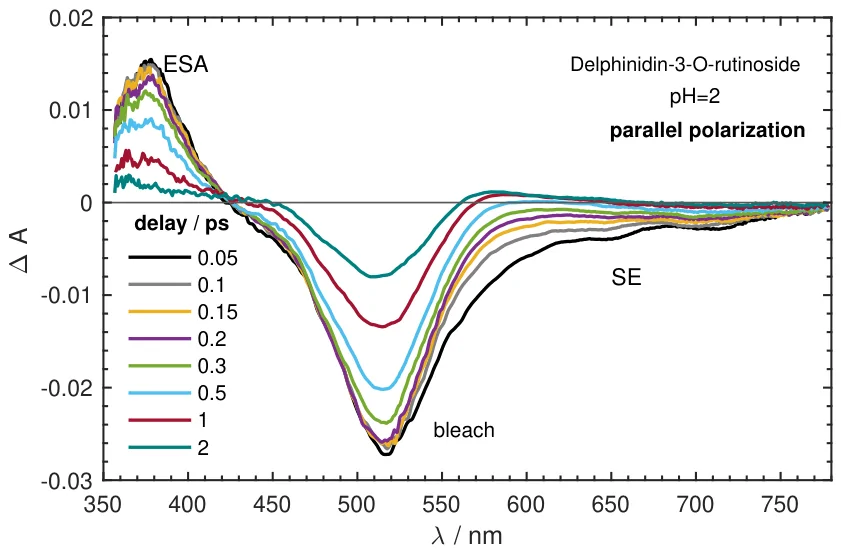
Femtosecond transient absorption of D3R at pH = 2 in aqueous solution upon excitation at 525 nm at the indicated pump-probe delays and parallel pump-probe polarization. Contributions from the stimulated emission (SE), excited state absorption (ESA), and bleaching signal (bleach) are indicated.

**Figure 3:**
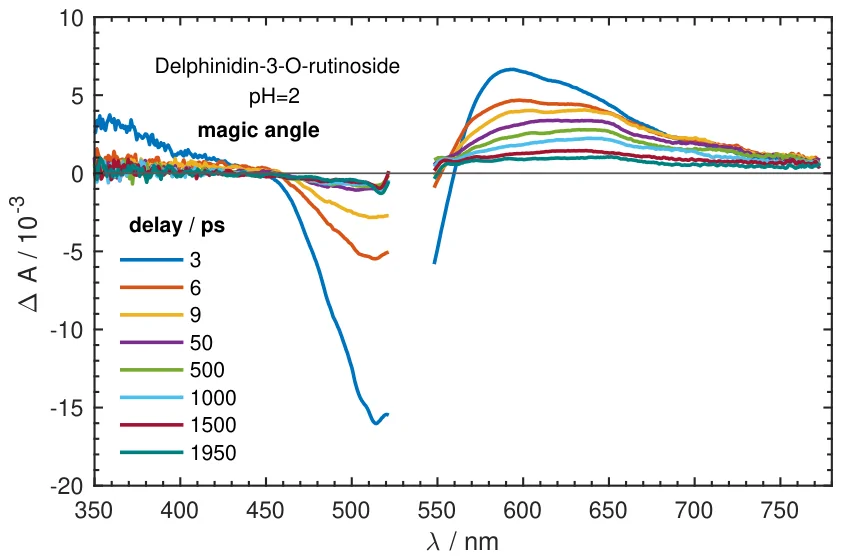
Transient absorption of D3R at pH = 2 in the picosecond-nanosecond timescale. Spectra obtained at magic angle polarization and at the indicated pump-probe delay times with slightly changed excitation conditions compared to Figure 2. The region around 525 nm is omitted, as large pump scattering fluctuations at later delays prevent reliable measurements in this spectral region.

**Figure 4:**
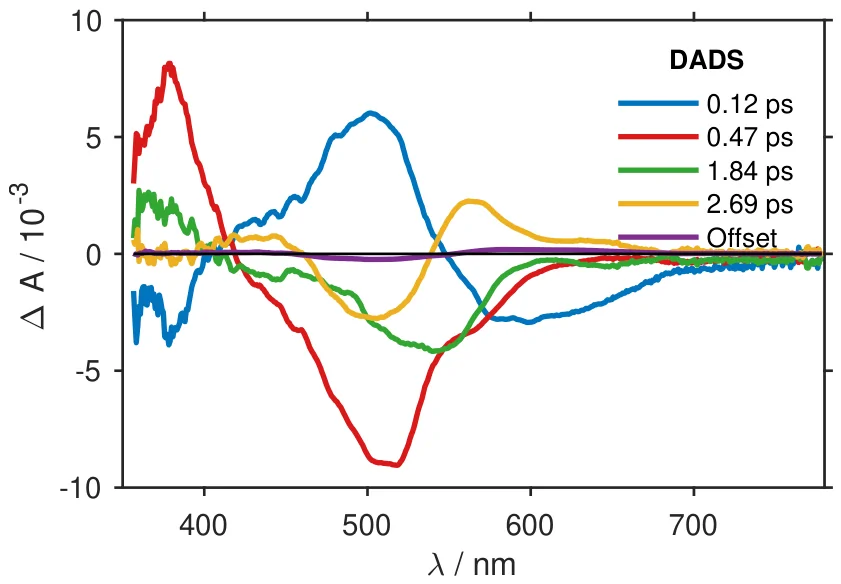
Dynamics of D3R: Decay associated difference spectra obtained from a global fit of the data presented in Figure 2. Decay times are indicated in the legend and are color coded. Since, the dataset was limited to 2 ps we observed a constant contribution in the global fit indicated by offset. Dynamics on longer delay times are presented in Figure 3.

**Figure 5:**
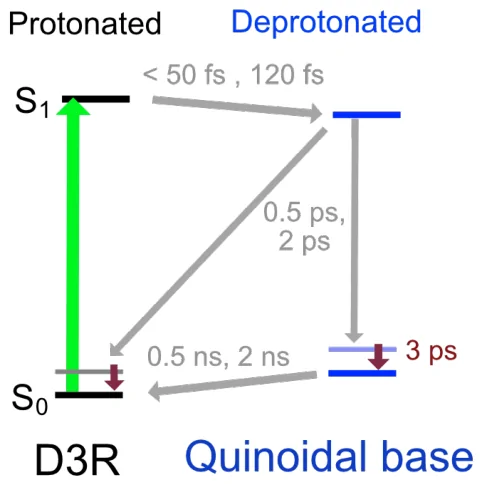
Reaction scheme upon photoexcitation of D3R.

## Data Availability

Dataset available on request from the authors.

## Supplementary Materials

The following supporting information can be downloaded at: https://www.mdpi.com/article/10.3390/molecules31203645/s1, Figure S1: Equilibraton time; Figure S2: Steady-State Fluorescence; Figures S3–S7: Global Analysis of the Transient Absorption Data; Figure S8: DADS of the slow dynamics; Figure S9: Comparison of D3R in H_2_O and D_2_O; Förster Cycle and Excited State Acidity. Refs. [26,27,31,32,33,34,35,36] are cited in Supplementary Materials.

## Abbreviations

The following abbreviations are used in this manuscript:

D3R	Delphinidin-3-rutinoside
D3R*	Electronic excited state of Delphinidin-3-rutinoside
D3R’	Vibrationally excited ground state of Delphinidin-3-rutinoside
QB	Quinoidal base
QB*	Electronic excited state of Quinoidal base
ESA	Excited state absorption
ES	Excited state
SE	Stimulated emission
