# Supplementary material for: Ultrafast, Non-Productive, Deprotonation of the Excited Flavylium Cation in Delphinidin-3-O-Rutinoside
Source: Molecules. 2026 Oct 13;31(20):3645. doi: 10.3390/molecules31203645 (PMC13707703; doi:10.3390/molecules31203645)
Supplement: Supplementary file 1 [file molecules-31-03645-s001.zip › molecules-4554717-supplementary.pdf]

# SUPPORTING INFORMATION

## Ultrafast, Non-Productive, Deprotonation of the Excited Flavylium Cation in Delphinidin-3-O-rutinoside

F. Bartonitz [1], J. Luis Pérez Lustres [1], and K. Heyne [1,*]

[1] Institut für Experimentalphysik, Freie Universität Berlin, Arnimallee 14, D-14195 Berlin (Germany)

[*] Correspondence: karsten.heyne@fu-berlin.de

# Equilibration time

The time interval between sample preparation and completion of the stationary absorption measurement was kept constant at 4 min for all experiments to ensure reproducible initial conditions. This consisted of approximately 2 min for sample transfer and 2 min for spectral acquisition. While no significant spectral changes were observed for D3R under strongly acidic conditions (pH 0–3), time-dependent changes occurred at higher pH values. These changes arise because, in addition to the rapid protonation and deprotonation reactions, slower hydration and subsequent ring-opening reactions govern the establishment of the pH-dependent multistate equilibrium of anthocyanins [35, 31]. Consequently, maintaining an identical timing between sample preparation and measurement ensured that all transient absorption experiments were performed under comparable initial conditions.

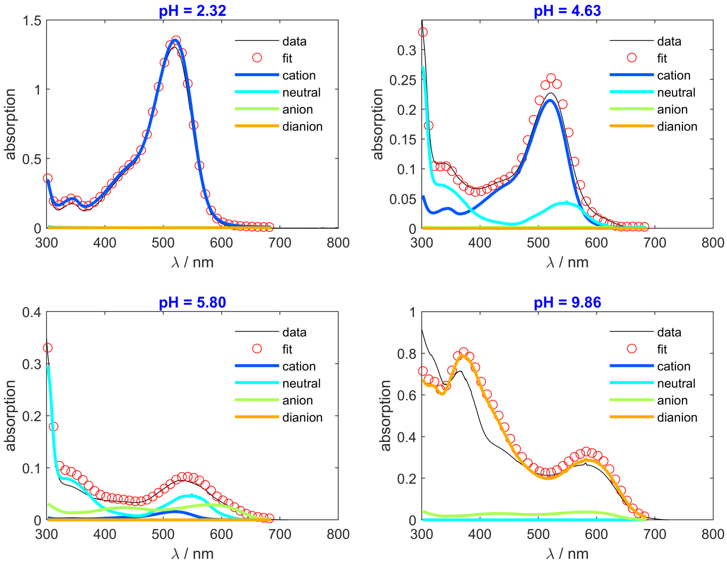


Figure S1: pH dependence of D3R linear absorption spectra and fits.

In Figure S1 we present experimental linear absorption spectra at four selected pH values (black lines). A simulated decomposition of the measured linear absorption spectrum (black line) into four different contributions: cationic form (blue line), neutral form(s) (cyan line), anionic form (green line), and another unknown form we call dianionic form (orange line) is shown. We performed a global analysis with these four components. The simulated data are shown in red circles (fit) together with the spectra of the individual contributions. It is clearly visible that at pH 2.32 only the cationic form is present, whereas at pH 4.63 the contribution from the neutral form

increases while the cationic form decreases. The overall decrease in oscillator strength is visible at pH 5.80. The dianionic form is dominant at pH 9.86.

# Steady-state Fluorescence

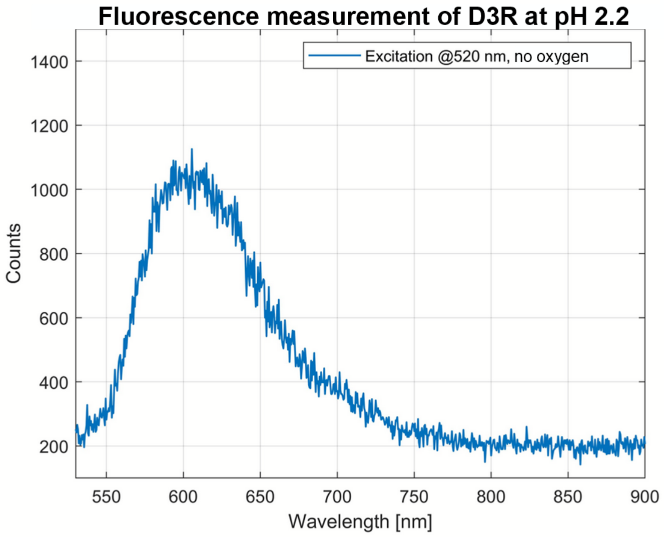


Figure S2: D3R fluorescence spectrum at pH 2.2 excited at 520 nm.

# Global Analysis of the Transient Absorption Data

The transient absorption (TA) datasets were analyzed by global fitting using a custom-written MATLAB routine. In contrast to independent fitting of individual kinetic traces, all probe wavelengths were analyzed simultaneously while sharing a common set of kinetic parameters. Only the amplitudes of the individual kinetic components were allowed to vary with probe wavelength. This approach assumes that the underlying photophysical processes are identical throughout the spectrum and differ only in their spectral contributions, thereby improving the robustness and uniqueness of the extracted kinetic parameters [32].

The measured transient absorption signal can be expressed as

$$\Delta A(\lambda, t) = \sum_{i=1}^{N} A_i(\lambda) K_i(t), \tag{S1}$$

where $A_i(\lambda)$ represents the wavelength-dependent amplitude (the decay-associated difference spectrum, DADS) of the $i$-th kinetic component and $K_i(t)$ denotes its corresponding temporal basis function.

The instrument response function (IRF) was approximated by a Gaussian function

$$G(t) = \exp\left[-\frac{(t-t_0)^2}{\sigma^2}\right], \tag{S2}$$

where $\sigma$ describes the Gaussian width and $t_0$ is the temporal overlap between pump and probe pulses.

The population dynamics were described by exponentially decaying components analytically convoluted with the Gaussian IRF,

$$K_i(t) = \exp\left(-\frac{t-t_0}{\tau_i} + \frac{\sigma^2}{4\tau_i^2}\right) \operatorname{erfc}\left(\frac{\sigma}{2\tau_i} - \frac{t-t_0}{\sigma}\right), \tag{S3}$$

where $\tau_i$ denotes the lifetime of the corresponding kinetic process and erfc is the complementary error function. This analytical solution corresponds to the convolution of a mono-exponential decay with a Gaussian instrument response function and is widely used in ultrafast spectroscopy [32].

To account for coherent artifacts around temporal overlap, the model additionally included the Gaussian function and its first four derivatives. These terms empirically describe coherent pump–probe interference and other ultrafast non-resonant contributions localized around time zero. Furthermore,

an error-function step was included to account for long-lived signal contributions extending beyond the experimental observation window.

For each iteration of the nonlinear optimization, the temporal basis matrix

$$\mathbf{K} = \begin{bmatrix} K_1(t_1) & \cdots & K_M(t_1) \\ \vdots & \ddots & \vdots \\ K_1(t_N) & \cdots & K_M(t_N) \end{bmatrix} \tag{S4}$$

was completely determined by the nonlinear parameters (Gaussian width, time-zero positions, and lifetimes). The wavelength-dependent amplitudes were subsequently obtained by solving the linear least-squares problem

$$\mathbf{A} = \mathbf{K}\backslash\mathbf{S}, \tag{S5}$$

where $\mathbf{S}$ denotes the experimental TA data matrix and $\mathbf{K}^{\dagger}$ is the Moore–Penrose pseudoinverse. In the MATLAB implementation this corresponds to the backslash operator (`A = K\S`), which computes the least-squares solution without explicitly forming the pseudoinverse [33].

The nonlinear optimization of the shared kinetic parameters was performed using MATLAB's `lsqnonlin` routine until convergence of the global residual. After convergence, the wavelength-dependent amplitudes were arranged into decay-associated difference spectra (DADS), providing the spectral signatures of the individual kinetic components.

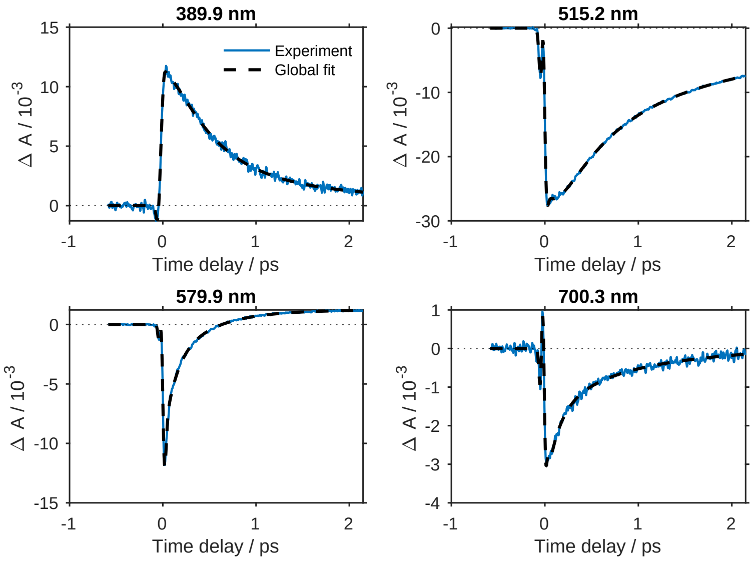


Figure S3: Selected transients and simulations from the global fit including the non-linear artefact at time zero on a short time-scale.

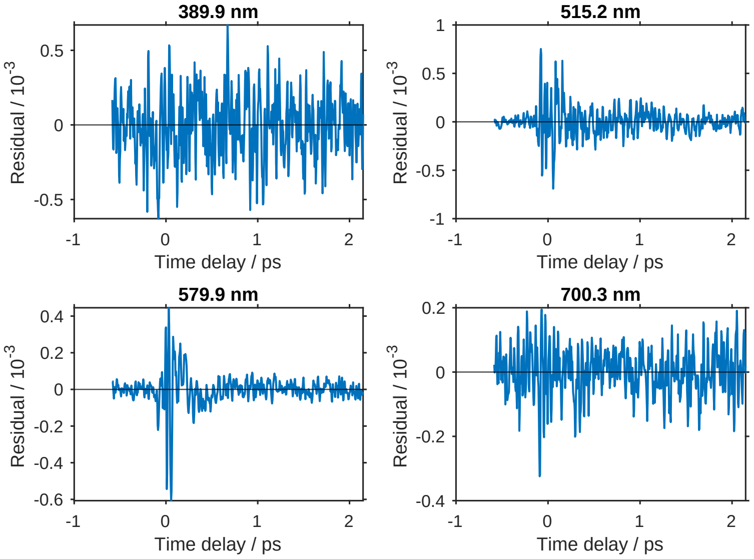


Figure S4: Residues of the transient data and simulation presented in Figure S3.

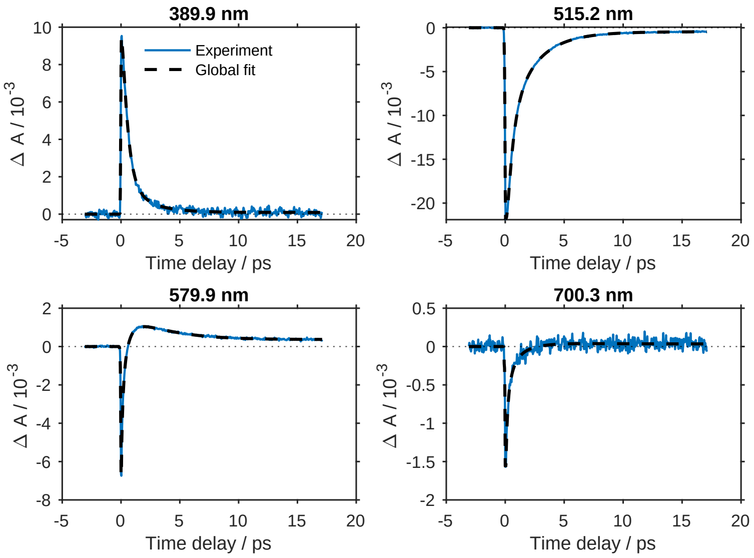


Figure S5: Selected transients and simulations from the global fit including the non-linear artefact at time zero on a longer time-scale.

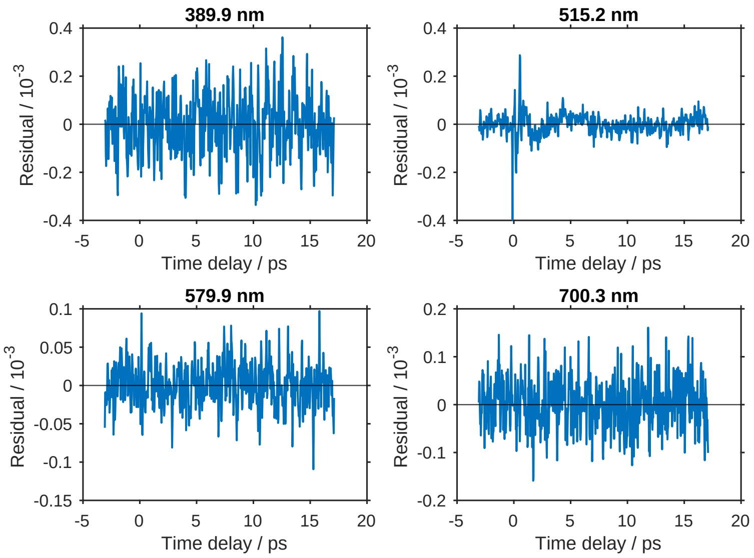


Figure S6: Residues of the transient data and simulation presented in Figure S5.

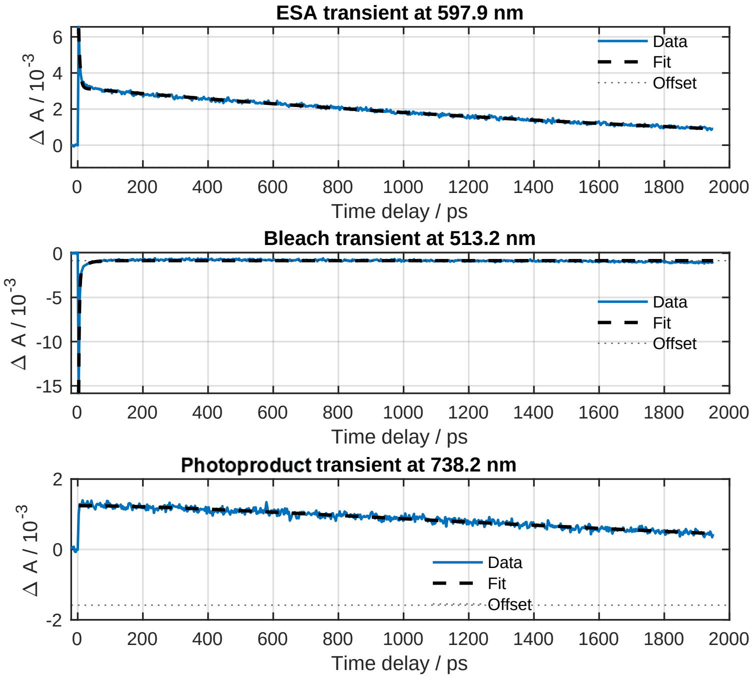


Figure S7: Selected transients and simulation from the global fit for the long time-scale.

# DADS of the slow dynamics

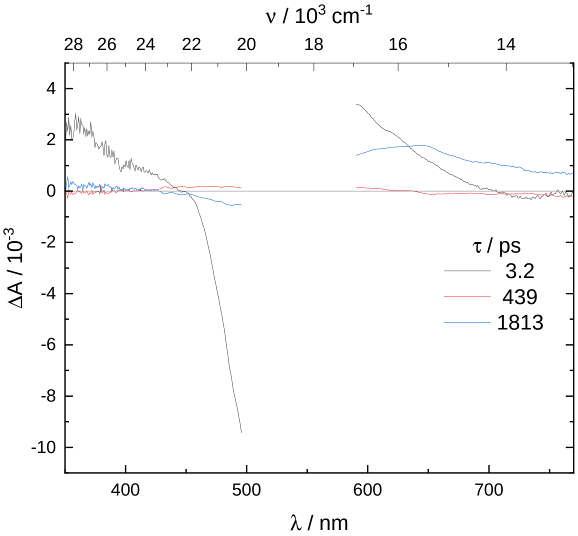


Figure S8: Decay associated difference spectra for the long time scale from a few picoseconds to nanoseconds.

# Comparison of D3R in $H_2O$ and $D_2O$

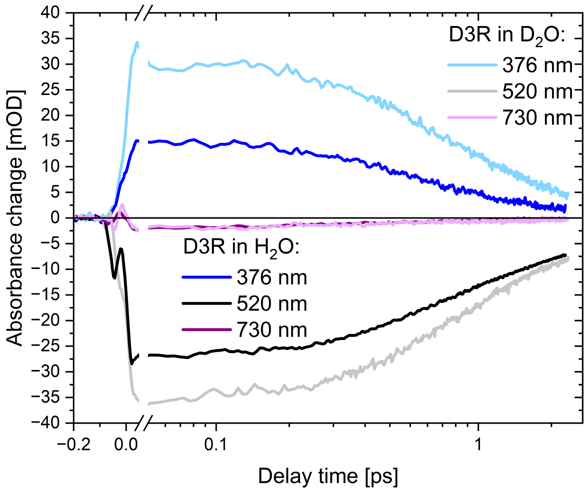


Figure S9: Transients of D3R dynamics in the cationic form in $H_2O$ (parallel polarized) and $D_2O$ (magic angle conditions). The corresponding transients measured at the same wavelengths show negligible differences in the dynamics. From these data we do not see any indications for an influence of H/D exchange on the ultrafast dynamics.

## Förster Cycle and Excited-State Acidity

The experimentally observed ultrafast dynamics are consistent with an excited-state proton transfer (ESPT) process. A qualitative estimate of the change in acidity upon photoexcitation can be obtained using the Förster cycle, which relates the ground-state acidity ($pK_{\mathrm{a}}$) to the excited-state acidity ($pK_{\mathrm{a}}^*$) through the difference in electronic transition energies of the protonated and deprotonated species [26, 27].

$$pK_{\mathrm{a}}^* = pK_{\mathrm{a}} - \frac{\Delta E}{2.303RT}, \tag{S6}$$

where $\Delta E$ is the difference between the electronic transition energies of the acid and its conjugate base, $R$ is the universal gas constant, and $T$ is the absolute temperature.

According to the Förster cycle, electronic excitation substantially modifies the acid–base equilibrium. For photoacids such as anthocyanins, excitation generally increases the acidity of the flavylium cation, resulting in a significantly lower excited-state acidity constant ($pK_{\mathrm{a}}^*$) than in the ground state. Consequently, proton transfer becomes thermodynamically more favorable immediately after photoexcitation, providing the driving force for ultrafast ESPT [26, 27].

It should be noted that the Förster equation provides a thermodynamic estimate of the excited-state acidity and does not describe the reaction kinetics. The experimentally observed sub-picosecond time constants therefore reflect the kinetic response of the system following excitation rather than the Förster equilibrium itself. Nevertheless, the calculated decrease in $pK_{\mathrm{a}}^*$ supports the assignment of the ultrafast transient components to excited-state proton transfer, while the subsequent picosecond dynamics are attributed to vibrational cooling, solvent relaxation, and structural reorganization of the excited-state population [34, 36].

## References

[26] Förster, T. Elektrolytische Dissoziation angeregter Moleküle. *Z. Elektrochem. Angew. Phys. Chem.* **1950**, *54*, 42–46. https://doi.org/10.1002/bbpc.19500540111.

[27] Weller, A. Outer and Inner Mechanism of Reactions of Excited Molecules. *Discuss. Faraday Soc.* **1959**, *27*, 28–33. https://doi.org/10.1039/DF9592700028.

[31] Pina, F.; Melo, M.J.; Laia, C.A.T.; Parola, A.M.; Lima, J.C. Chemistry and applications of anthocyanins: A review. *Chem. Soc. Rev.* **2012**, *41*, 869–908.

[32] van Stokkum, I.H.M.; Larsen, D.S.; van Grondelle, R. Global and target analysis of time-resolved spectra. *Biochim. Biophys. Acta* **2004**, *1657*, 82–104.

[33] Golub, G.H.; Van Loan, C.F. *Matrix Computations*, 4th ed.; Johns Hopkins University Press: Baltimore, MD, USA, 2013.

[34] Kiefer, P.M.; Hynes, J.T. Nonlinear Free Energy Relations for Adiabatic Proton Transfer Reactions in a Polar Environment. II. Inclusion of the Hydrogen Bond Vibration. *J. Phys. Chem. A* **2002**, *106*, 1850–1861. https://doi.org/10.1021/jp013425w.

[35] Brouillard, R.; Dubois, J.E. Mechanism of the Structural Transformations of Anthocyanins in Acidic Media. *J. Am. Chem. Soc.* **1977**, *99*, 1359–1364. https://doi.org/10.1021/ja00447a039.

[36] Agmon, N. The Grotthuss Mechanism. *Chem. Phys. Lett.* **1995**, *244*, 456–462. https://doi.org/10.1016/0009-2614(95)00905-J.
